# Human Claudin-Derived Peptides Block the Membrane Fusion Process of Zika Virus and Are Broad Flavivirus Inhibitors

**DOI:** 10.1128/spectrum.02989-22

**Published:** 2022-08-30

**Authors:** Jim Zoladek, Julien Burlaud-Gaillard, Maxime Chazal, Sophie Desgraupes, Patricia Jeannin, Antoine Gessain, Nathalie Pardigon, Mathieu Hubert, Philippe Roingeard, Nolwenn Jouvenet, Philippe V. Afonso

**Affiliations:** a Unité Épidémiologie et Physiopathologie des Virus Oncogènes, Institut Pasteur, Université Paris Cité, CNRS UMR 3569, Paris, France; b Inserm U1259 MAVIVH, Université de Tours and CHRU de Tours, Tours, France; c Plate-Forme IBiSA de Microscopie Electronique, Université de Tours and CHRU de Tours, Tours, France; d Unité Signalisation Antivirale, Institut Pasteur, Université Paris Cité, CNRS UMR 3569, Paris, France; e Groupe Arbovirus, Unité Environnement et Risques Infectieux, Institut Pasteur, Université Paris Cité, Paris, France; f Unité Virus et Immunité, Institut Pasteur, Université Paris Cité, CNRS UMR 3569, Paris, France; David Geffen School of Medicine at UCLA

**Keywords:** antimicrobial peptides, claudin, flavivirus, Zika

## Abstract

Zika virus (ZIKV) is a mosquito-borne flavivirus that emerged in the Pacific islands in 2007 and spread to the Americas in 2015. The infection remains asymptomatic in most cases but can be associated with severe neurological disorders. Despite massive efforts, no specific drug or vaccine against ZIKV infection is available to date. Claudins are tight-junction proteins that favor the entry of several flaviviruses, including ZIKV. In this study, we identified two peptides derived from the N-terminal sequences of claudin-7 and claudin-1, named CL7.1 and CL1.1, respectively, that inhibited ZIKV infection in a panel of human cell lines. Using cell-to-cell fusion assays, we demonstrated that these peptides blocked the ZIKV E-mediated membrane fusion. A comparison of the antiviral efficacy of CL1.1 and CL7.1 pointed to the importance of the peptide amphipathicity. Electron microscopic analysis revealed that CL1.1 altered the ultrastructure of the viral particles likely by binding the virus lipid envelope. However, amphipathicity could not fully explain the antiviral activity of CL1.1*. In silico* docking simulations suggested that CL1.1 may also interact with the E protein, near its stem region. Overall, our data suggested that claudin-derived peptides inhibition may be linked to simultaneous interaction with the E protein and the viral lipid envelope. Finally, we found that CL1.1 also blocked infection by yellow fever and Japanese encephalitis viruses but not by HIV-1 or SARS-CoV-2. Our results provide a basis for the future development of therapeutics against a wide range of endemic and emerging flaviviruses.

**IMPORTANCE** Zika virus (ZIKV) is a flavivirus transmitted by mosquito bites that have spread to the Pacific Islands and the Americas over the past decade. The infection remains asymptomatic in most cases but can cause severe neurological disorders. ZIKV is a major public health threat in areas of endemicity, and there is currently no specific antiviral drug or vaccine available. We identified two antiviral peptides deriving from the N-terminal sequences of claudin-7 and claudin-1 with the latter being the most effective. These peptides block the envelope-mediated membrane fusion. Our data suggested that the inhibition was likely achieved by simultaneously interacting with the viral lipid envelope and the E protein. The peptides also inhibited other flaviviruses. These results could provide the basis for the development of therapies that might target a wide array of flaviviruses from current epidemics and possibly future emergences.

## INTRODUCTION

Many flaviviruses have been at the core of emergencies in recent years (1). The most recent is the Zika virus (ZIKV), which emerged on Yap Island in 2007, in French Polynesia in 2013, and in the Americas in 2015. These outbreaks have evidenced that ZIKV infection can cause severe neurological disorders, including Guillain-Barré syndrome (2), congenital microcephaly (3), meningoencephalitis (4), and myelitis (5).

Other flaviviruses, such as yellow fever virus (YFV), Japanese encephalitis virus (JEV), and West Nile virus (WNV), are frequently associated with epidemics. While there are approved vaccines to prevent some of these infections, there is currently no vaccine available against ZIKV infection. Furthermore, there is no specific antiviral drug treatment for these infections. The development of a pan-flavivirus treatment is necessary for treating current epidemics and future emergencies (1).

Peptide-based treatments have gained much attention in recent years as these molecules may constitute potent and safe antiviral drugs (6). Over the past years, several peptides with anti-ZIKV properties have been described. Most have been designed from the sequences of flavivirus envelope (E) proteins. Their mechanisms of action are quite diverse. Some anti-ZIKV peptides disrupt the integrity of the viral particles, therefore preventing infection *in vitro* (7) and *in vivo* (8–10), while others have been shown to interact with viral proteins and interfere with their functions *in vitro* (11). In many cases, their mechanism remains unclear (12–14).

We have recently demonstrated that claudin-7 was required for optimal ZIKV replication in human endothelial cells (15). We showed that claudin-7 is involved in a postinternalization and pre-replication step, likely during viral sorting in endosomes where the fusion with endosomal membrane occurs (15). Consequently, we wondered whether targeting the interactions between claudin-7 and ZIKV could be used as a strategy to reduce ZIKV infection in human cells. The use of antibodies directed against claudin-7 was not possible. Indeed, the commercially available antibodies do not recognize the extracellular loops of claudin-7, where the binding domain to ZIKV is likely located since the extracellular loops of claudin-1 have been previously reported to interact with Dengue virus (DENV) (16, 17). Moreover, claudin-7 expression levels in endothelial cells are too low to be detected with antibodies (15, 18). Thus, antibodies would probably not disrupt the interaction between ZIKV and claudin-7. We, therefore, designed peptides based on the claudin-7 sequence to block the interaction.

We found that peptides derived from the N-terminal sequences of claudin-7 and claudin-1 exhibited antiviral properties against ZIKV in several human cell lines. Both peptides blocked E-mediated cell-to-cell membrane fusion, with the claudin-1-derived peptide being the most effective. These peptides did not correspond to the extracellular domains of the claudins. Therefore, we speculated that the inhibition was achieved by directly interfering with the interaction between the cellular claudin and the viral prM/E protein. We aimed at understanding the mechanism of such inhibition and focused primarily on CL1.1, the most effective peptide. We found that CL1.1 did not alter virus binding to host membranes or internalization in the target cells. We found that CL1.1 altered the ultrastructure of the viral particles, which was presumably the consequence of peptides interacting with the virus lipid envelope. Indeed, we found that peptide amphipathicity was crucial for efficient antiviral activity. However, amphipathicity was not sufficient to inhibit ZIKV infection as a control peptide with similar amphipathicity, CL7.4, did not inhibit ZIKV infection. *In silico* docking, predictions were performed and suggested that the peptides may also interact with the stem domain of the E protein. Our data suggested that the simultaneous interaction of the peptide with the lipid envelope and the E protein may inhibit virus fusion by preventing the lipid envelope curvature changes and/or the conformational changes of the E protein. We finally found that CL1.1 also blocked JEV and YFV infection but not unrelated enveloped viruses, such as HIV-1 and SARS-CoV-2. These findings provided a novel strategy for developing antiviral peptides that could potentially target a wide range of flaviviruses.

## RESULTS

### Claudin N-terminus-derived peptides CL1.1 and CL7.1 inhibited ZIKV replication.

We designed 16 overlapping 18-mer peptides covering the entire sequence of human claudin-7 (S1 Table in Supplemental File 1). Eight peptides could not be synthesized and were therefore abandoned. We tested the capacity of the remaining peptides to inhibit ZIKV infection in hCMEC/D3 cells, a human cerebral microvascular endothelial cell line, in which claudin-7 favors ZIKV replication (15).

**FIG 1:**
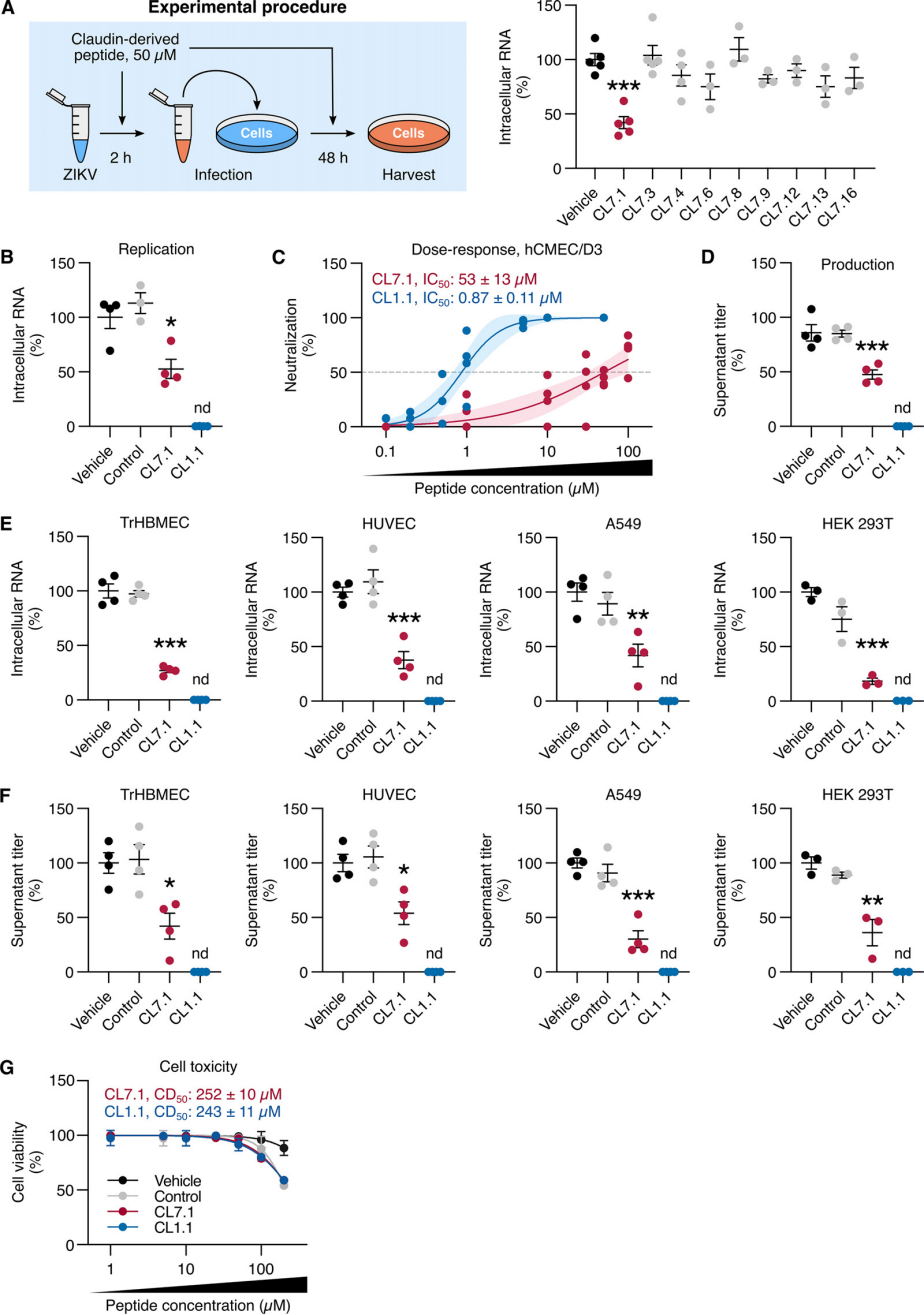
Claudin-derived peptides block ZIKV infection in different cell types. (A) ZIKV inoculum (5 × 10^5^ PFU/mL, equivalent to an MOI of 1) was treated with 50 μM claudin-7-derived 18-mer peptides or an equivalent volume of vehicle (DMSO) for 1 h. These inocula were used to infect hCMEC/D3 cells for 2 h, which were subsequently washed and kept in a fresh medium containing peptides for 48 h. Viral replication was assessed by RT-qPCR on intracellular RNA. (B) The efficiency of 50 μM CL7.1 or CL1.1 to block ZIKV infection (MOI of 1) was assessed in hCMEC/D3. Viral replication was assessed by RT-qPCR on intracellular RNA (compared to control peptide CL7.4, or vehicle [DMSO] alone). (C) Dose-dependent effect of CL7.1 and CL1.1 on ZIKV infection (MOI of 1) in hCMEC/D3. The line represents the fitted dose-response model for each peptide and the light area represents the 95% confidence interval of the model. The dashed line represents 50% of viral neutralization. The calculated IC_50_ is indicated. R^2^ values were 0.9 and 0.7 for CL1.1 and CL7.1, respectively. (D) Supernatants of the cells in (B) were titrated on Vero E6 cells to assess viral production. (E) The efficiency of 50 μM CL7.1 and CL1.1 to inhibit ZIKV infection (MOI of 1) was assessed in TrHBMEC, HUVEC, A549, and HEK 293T cells Viral replication was assessed by RT-qPCR on intracellular RNA (compared to 50 μM control peptide CL7.4, or vehicle alone). (F) Supernatants of the cells in (E) were titrated on Vero E6 cells to assess viral production. (G) A549 cells were treated with increasing concentrations of CL1.1, or CL7.1, or control CL7.4 for 48 h. Cell viability was measured with an MTT assay (compared to control peptide CL7.4 or vehicle alone). The calculated CD_50_ for CL1.1 is indicated. Data are presented as individual biological replicates and mean ± SEM (A to F) or mean ± SEM (G, *n *= 3 biological replicates); nd, not detected; ***, *P ≤ *0.001; **, *P ≤ *0.01; *, *P ≤ *0.05 (One-way ANOVA followed by Dunnett’s *post hoc*).

ZIKV inocula from the African lineage (5 × 10^5^ PFU/mL) were treated with 50 μM of the different peptides for 1 h and then led to the adsorption on hCMEC/D3 cells for 2 h (multiplicity of infection [MOI] of 1). Cells were washed and maintained in a fresh medium containing 50 μM peptides for 48 h, following a screening procedure previously developed to identify anti-HCV peptides (19). RT-qPCR analyses were performed to determine the levels of intracellular viral RNA, a readout for ZIKV replication. We found that a peptide derived from the N-terminus of claudin-7 (Fig. S1 in Supplemental File 1), termed CL7.1, induced a 2-fold reduction of viral RNA levels compared to untreated cells (Fig. 1A). Treatment with the other claudin-7-derived peptides had no significant impact on ZIKV infection (Fig. 1A). Of note, the antiviral activity of CL7.1 peptide was also detected in the absence of pretreatment, when virus and peptide were added simultaneously to the cell culture (Fig. S2 in Supplemental File 1).

Our data were in line with a previous study showing that a peptide derived from the N-terminus of claudin-1 (named herein CL1.1) exhibited antiviral activity against HCV infection of Huh7.5.1 cells (19). We tested the activity of CL1.1 against ZIKV infection and found that CL1.1 exhibited stronger antiviral activity than CL7.1. Intracellular viral RNA levels were below the threshold of detection in hCMEC/D3 cells treated with 50 μM CL1.1 (Fig. 1B). Inhibition was dose-dependent, with the IC_50_ values around 53 ± 13 μM for CL7.1 and 0.87 ± 0.11 μM for CL1.1 (Fig. 1C).

Infectious particle production was assessed in the supernatants of infected hCMEC/D3 cells. Consistent with the replication data (Fig. 1B), viral production was unaffected by 50 μM control CL7.4 (see Materials and Methods for justification), significantly reduced upon treatment with 50 μM CL7.1, and below the detection limit of the assay in the presence of 50 μM CL1.1 (Fig. 1D).

To assess whether the treatment was cell type-dependent, we tested the impact of the peptides on ZIKV infection of two human endothelial cell lines – umbilical vein endothelial cells (HUVEC) and bone marrow endothelial cells (TrHBMEC) – and two human epithelial cell lines – alveolar basal cells (A549) and embryonic kidney cells (HEK 293T). ZIKV replication and production were significantly reduced in the presence of 50 μM CL7.1 in the four cell lines we tested, and undetectable in the presence of 50 μM CL1.1 (Fig. 1E and F). A control peptide (50 μM CL7.4) did not inhibit infection in any tested cell lines (Fig. 1E and F).

To ensure that the inhibition of viral replication and production in the presence of claudin-derived peptides were not biased by peptide-induced cell toxicity, we performed MTT assays on A549 cells treated with increasing concentrations of CL1.1, CL7.1, or CL7.4 (the control peptide) for 48 h. The assay showed no significant toxicity up to 100 μM for each peptide. The cytotoxic dose 50 (CD_50_) was estimated at 250 ± 10 μM for every peptide (Fig. 1G). The treatment of differentiated monolayers of Caco-2 epithelial cells with 50 μM CL1.1 did not alter the electric transepithelial resistance, confirming the absence of toxicity (Fig. S3 in Supplemental File 1). Overall, our results suggested that the antiviral activity of CL1.1 was not due to peptide-induced cell toxicity.

These results indicated that both CL1.1 and CL7.1 displayed anti-ZIKV activity regardless of the target cell type, with CL1.1 being more potent. Hence, we prioritized our study on CL1.1.

### CL1.1 neutralized ZIKV particles directly.

**FIG 2:**
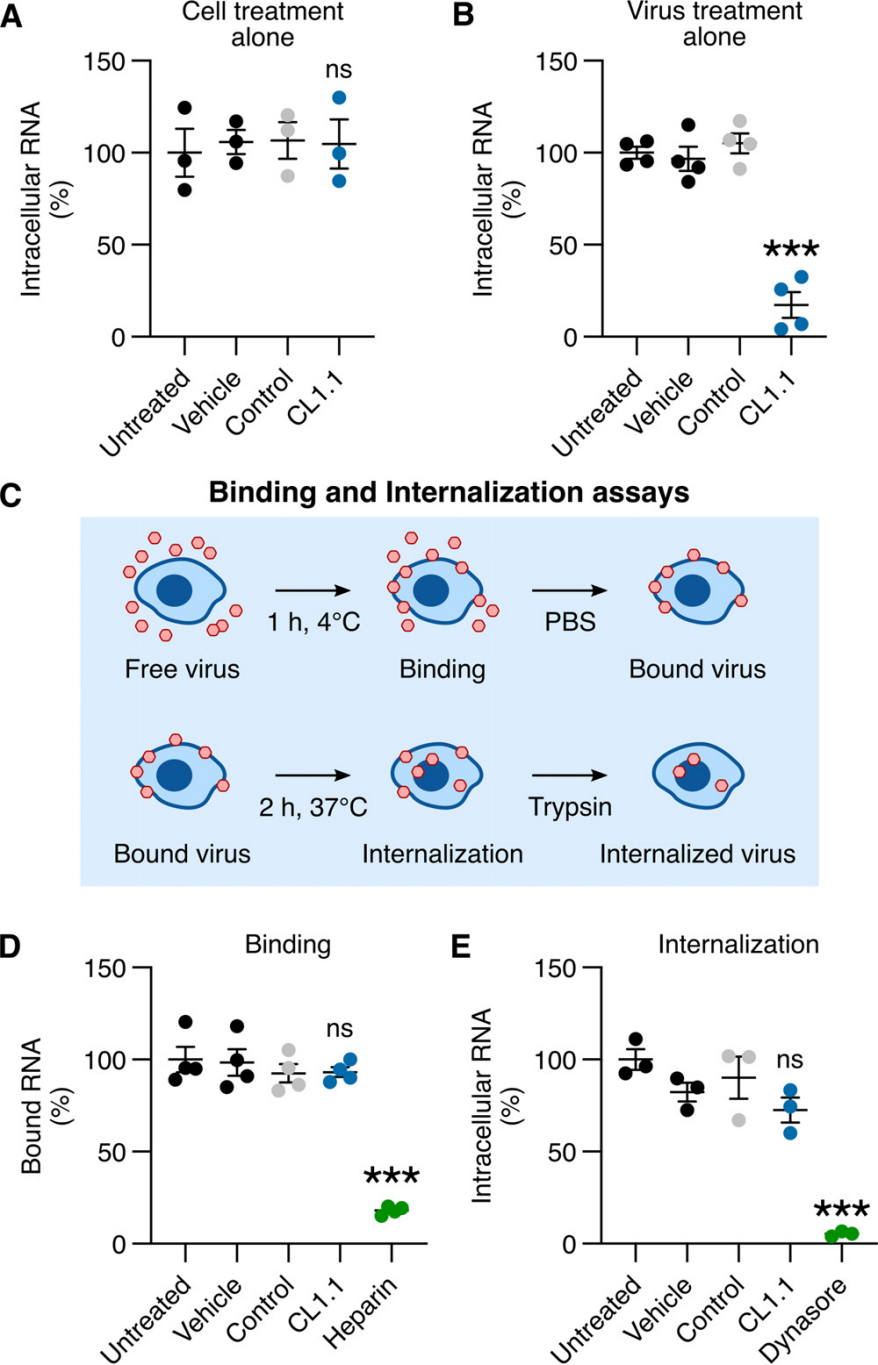
Claudin-derived peptides blocked ZIKV at a post-internalization step without altering cellular functions. (A) hCMEC/D3 cells were treated with 10 μM CL1.1 for 1 h and extensively washed with PBS to remove excess peptides. The cells were then infected with ZIKV (MOI of 1) for 48 h. Viral replication was assessed by RT-qPCR on intracellular RNA (compared to control peptide CL7.4 or vehicle alone). (B) ZIKV (equivalent MOI of 30) was treated with 10 μM CL1.1 for 1 h. The treated inocula were then diluted 30-times and used to infect hCMEC/D3 cells for 48 h (MOI of 1). Viral replication was assessed by RT-qPCR on intracellular RNA (compared to control peptide CL7.4 or vehicle alone). (C) Experimental procedure of the binding/internalization assay. ZIKV (MOI of 10) was adsorbed in hCMEC/D3 cells for 1 h at 4°C. The cells were then extensively washed with cold PBS to remove unbound viruses. Viral binding was analyzed at this stage by RT-qPCR. The cells were then incubated for 2 h at 37°C to induce internalization, washed with PBS, and treated with Trypsin to remove viruses that remained at the cell surface. Viral internalization was analyzed at this stage by RT-qPCR. (D) ZIKV was treated with 10 μM CL1.1 or 200 μg/mL Heparin for 1 h before the binding assay. Virus bound to cells was assessed by RT-qPCR (compared to 10 μM control peptide CL7.4, or vehicle alone). (E) ZIKV was treated with 10 μM CL1.1 or 100 μM Dynasore for 1 h before the internalization assay, fresh peptide and Dynasore were added during the internalization process. Viral internalization was assessed by RT-qPCR (compared to control peptide CL7.4 or vehicle alone). Data are presented as individual biological replicates ± SEM; ***, *P ≤ *0.001; *, *P ≤ *0.05; ns, *P* > 0.05 (one-way analysis of variance [ANOVA] followed by Dunnett’s *post hoc*).

We wondered whether CL1.1 acted via an interaction with the target cell or the viral particle. We found that pretreatment of hCMEC/D3 cells with 10 μM CL1.1 (which did suffice to significantly inhibit ZIKV in Fig. 1B) did not affect cell susceptibility to ZIKV. Viral replication was comparable between untreated cells and cells treated with CL1.1 or control peptides (Fig. 2A).

We incubated concentrated ZIKV stocks (1.5 × 10^7^ PFU/mL) with 10 μM CL1.1 or control peptide for 1 h. The viral inoculum was then diluted by a factor of 30 so that the final concentrations of peptides were ineffective against ZIKV infection (see Fig. 1C). The hCMEC/D3 cells were then infected with the treated and diluted inocula. Forty-eight hours after infection, intracellular viral RNA levels were reduced by 80% upon treatment with CL1.1 (Fig. 2B).

These results suggested that CL1.1 inhibited viral infection by interacting with ZIKV particles rather than the target cell. This was consistent with the fact that the peptide-mediated viral inhibition was independent of the target cell type (Fig. 1E and F).

### CL1.1 did not alter early ZIKV interactions with the cell.

ZIKV inhibition could be achieved by preventing viral adsorption to or internalization in the cells. To investigate these steps of the ZIKV cycle, we used viral binding and internalization assays (Fig. 2C) that we previously established (15).

We incubated a ZIKV inoculum (5 × 10^6^ PFU/mL) with 10 μM CL1.1 or control peptide (CL7.4) for 1 h and let it adsorb on hCMEC/D3 cells for 1 h at 4°C (MOI of 10). Viral binding was assessed by RT-qPCR after the removal of unbound viruses by PBS wash. We found that treatment with CL1.1 did not alter the binding of ZIKV to hCMEC/D3 cells (Fig. 2D). Heparin, a glycosaminoglycan known to interfere with viral binding to the cell (20), was used as a positive control and led to an 80% reduction of viral binding (Fig. 2D).

Following binding, the cells were incubated for 2 h at 37°C in a fresh medium containing peptides to enable endocytosis. Viral internalization was assessed by RT-qPCR after the removal of the viral particles that remained at the cell surface using trypsin. Treatment with 10 μM CL1.1 did not alter ZIKV internalization in hCMEC/D3 cells (Fig. 2E). Dynasore, an inhibitor of dynamin that blocks ZIKV internalization (21), was used as a positive control and led to a 95% reduction of viral internalization (Fig. 2E).

The data showed that CL1.1 blocked ZIKV infection neither at adsorption nor internalization steps but they should at a later step of the interaction with the cell, for example, by inhibiting the fusion of the viral envelope with the endosomes.

### CL1.1 and CL7.1 blocked the pH-dependent fusion step of the ZIKV cycle.

An approach using ZIKV-induced Aedes albopictus C6/36 syncytium formation has been previously used to test viral fusion inhibitors (22, 23). We took advantage of this strategy to study the impact of claudin-derived peptides on ZIKV E-mediated fusion.

**FIG 3:**
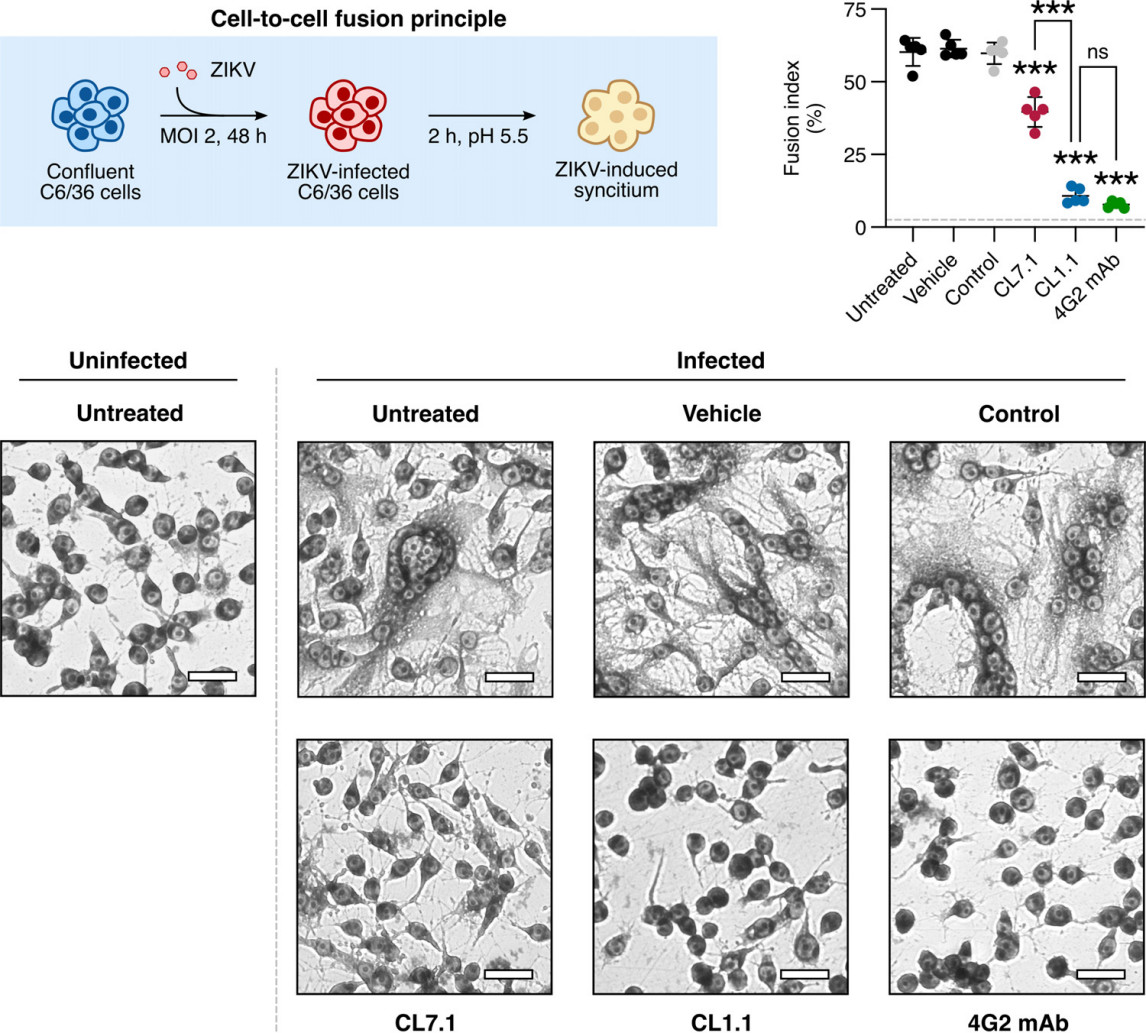
CL1.1 and CL7.1 blocked ZIKV-induced cell-to-cell fusion in C6/36 cells. C6/36 cells were infected with ZIKV (MOI of 2) for 48 h before the experiment. The cells were then treated with 10 μM CL1.1, 50 μM CL7.1, or 50 μM of the control peptide (i.e., CL7.4), or 500 ng/mL 4G2 MAb for 1 h. Cell-to-cell fusion was induced for 2 h by medium acidification, and the cells were fixed, stained with Giemsa, and analyzed with bright-field microscopy (compared to control peptide CL7.4 or vehicle alone). The fusion index was calculated using the formula: 1 – (number of cytoplasms/number of nuclei) in fields of 200 ± 50 nuclei. The dashed line represents the fusion index of uninfected cells. Representative images of the cell-to-cell fusion are shown on the right. Scale: 25 μm. Data are presented as individual biological replicates and mean ± SEM; ***, *P ≤ *0.001; ns, *P* > 0.05 (one-way ANOVA followed by Dunnett’s *post hoc*). Uncropped source pictures are available in Fig. S7 in Supplemental File 1.

C6/36 cells were infected with ZIKV for 48 h (MOI of 2), and upon acidification of the culture medium, cell-to-cell fusion occurred and syncytia were observed. Treatment of the inoculum with 500 ng/mL of the monoclonal antibody 4G2, which interacts with the E protein fusion loop (24), led to a reduction of more than 80% of cell-to-cell fusion. Fusion was not affected by the presence of DMSO (the vehicle) or the control peptide (50 μM C7.4) (Fig. 3). In the presence of 10 μM CL1.1 or 50 μM CL7.1, syncytium formation was reduced by 80% and 40%, respectively (Fig. 3). The lower inhibition level of cell-to-cell fusion achieved with CL7.1 as opposed to CL1.1 was consistent with its lower antiviral potency (Fig. 1 and 3).

These results suggested that CL1.1 and CL7.1 inhibited ZIKV infection by blocking the pH-dependent E-mediated membrane fusion.

### Differences in amphipathicity correlated with differences in antiviral potency between CL1.1 and CL7.1.

Although CL1.1 and CL7.1 differ by only 4 amino acids, their antiviral potency was significantly different (Fig. 1 to 3). We sought to determine which amino acids were responsible for this difference.

**FIG 4:**
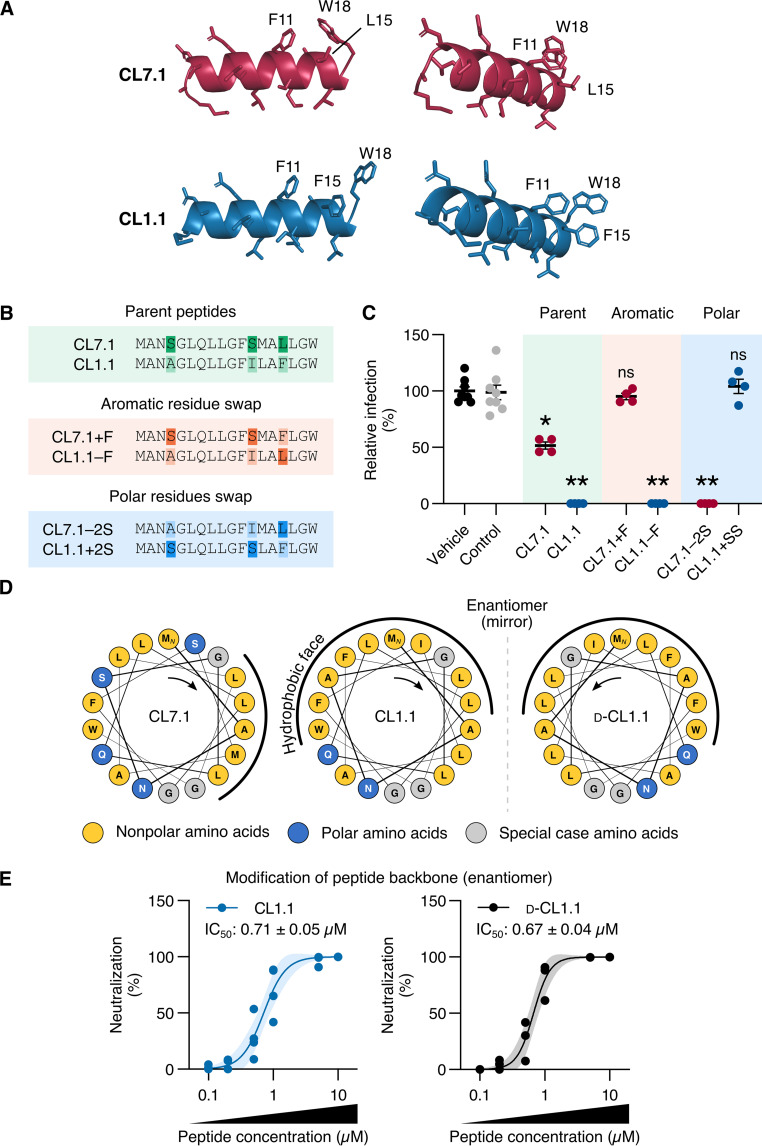
Amphipathicity was involved in the differences in potency between claudin-derived peptides. (A) The tridimensional structure of CL7.1 (red) and CL1.1 (blue) was predicted using PEP-FOLD3. Three amino acids of interest at positions 11, 15, and 18 are indicated. (B) Sequences of the parental and modified peptides. The swapped amino acids are highlighted. (C) ZIKV (MOI of 1) was treated with 50 μM of the modified peptides described in (B) for 1 h. These treated inocula were used to infect hCMEC/D3 cells for 2 h, unbound viruses were washed away, and infected cells were kept in a fresh medium containing peptides for 48 h. Viral production was assessed by titrating the supernatants (compared to control peptide CL7.4 or vehicle alone). (D) Helical wheel projections of CL7.1, CL1.1, and d-CL1.1. Left or right orientations of the helixes are indicated with an arrow and the hydrophobic faces of each peptide are shown with a black line. (E) Dose-dependent effect of CL1.1 and d-CL1.1 on ZIKV infection of Vero E6 cells (MOI of 1, Brazil/2016/INMI1 strain). The dark line represents the fitted dose-response model for each peptide and the light area represents the 95% confidence interval of the model. The calculated IC_50_ is indicated. Data are presented as individual biological replicates and mean ± SEM; **, *P ≤ *0.01; *, *P ≤ *0.05; ns, *P* > 0.05 (Kruskal-Wallis ANOVA followed by Dunn’s *post hoc*).

We used PEP-FOLD3, a peptide-oriented tertiary structure prediction software (25), to predict the structure of both peptides. According to the software, both peptides form alpha helices (Fig. 4A), which was consistent with the fact that these peptides have been designed on the first alpha helix of claudin-1 and claudin-7 (Fig. S1 in Supplemental File 1).

Of these predicted structures, we found that CL1.1 presented three aromatic amino acids that were spatially aligned (i.e., F11, F15, W18), whereas CL7.1 only had two (i.e., F11, W18) that could play a role in the antiviral effect (Fig. 4A). We tested the impact of swapping the amino acid in position 15 on antiviral potency of the peptides (Fig. 4B and C). ZIKV inocula were treated with 50 μM parental or modified peptides for 1 h and used to infect hCMEC/D3 cells. Viral production was determined by supernatant titration at 48 h postinfection. While the resulting CL7.1+F lost its antiviral potency, CL1.1–F retained its antiviral activity (Fig. 4C). Thus, although the aromatic amino acid at position 15 had some antiviral function, it was not the primary driver of antiviral activity.

Using HELIQUEST, a helix analysis software (26), we found that CL1.1 was amphipathic with a large continuous hydrophobic face (Fig. 4D). The calculated amphipathicity value of CL1.1 (μH) was 0.3 (27). We found that CL7.1 was slightly less amphipathic, the calculated μH was 0.2, and had a smaller hydrophobic face (Fig. 4D). This was the consequence of two polar serine residues (i.e., S4, S12) that were absent in CL1.1 (Fig. 4B). Therefore, we tested the impact of swapping residues S4 and S12 in CL7.1 with the corresponding nonpolar residues present in CL1.1 (i.e., A4 and I12) and vice-versa (Fig. 4B and C). As described above, we tested the impact of the modified peptides on ZIKV infection (at 50 μM). The removal of the two serines from CL7.1 (CL7.1−2S) increased the inhibition capacity of the peptide to a level comparable to that achieved with CL1.1. In contrast, the introduction of serines into the sequence of CL1.1 (CL1.1+2S) abolished its inhibition potency (Fig. 4C).

Of note, the control peptide (CL7.4, see Materials and Methods) displayed similar properties to that of CL1.1. It formed an amphipathic alpha helix with a calculated μH of 0.28 (Fig. S4 in Supplemental File 1).

If amphipathicity were to play a major role in the antiviral activity of the peptides, the d-enantiomer of CL1.1 should exhibit antiviral properties similar to that of the l-enantiomer. A d-enantiomer of an alpha helix forms a mirrored left-handed helix (Fig. 4D) but retains the same physicochemical properties (28). We found that the antiviral activity of d-CL1.1 against ZIKV infection of Vero E6 cells was identical to that of CL1.1 (Fig. 4E). The calculated IC_50_ values were 0.71 ± 0.05 μM and 0.67 ± 0.04 μM for CL1.1 and d-CL1.1, respectively (Fig. 4E).

Taken together, our results indicated that the difference in the anti-ZIKV efficacy between CL1.1 and CL7.1 correlated with amphipathicity.

### CL1.1 altered ZIKV particle ultrastructure without compromising viral integrity.

In some cases, the antiviral activity of amphipathic peptides has been associated with the capacity of the peptides to disrupt viral lipid envelopes or induce virus aggregation (8–10, 29). Therefore, we investigated potential peptide-induced alterations in ZIKV ultrastructure.

**FIG 5:**
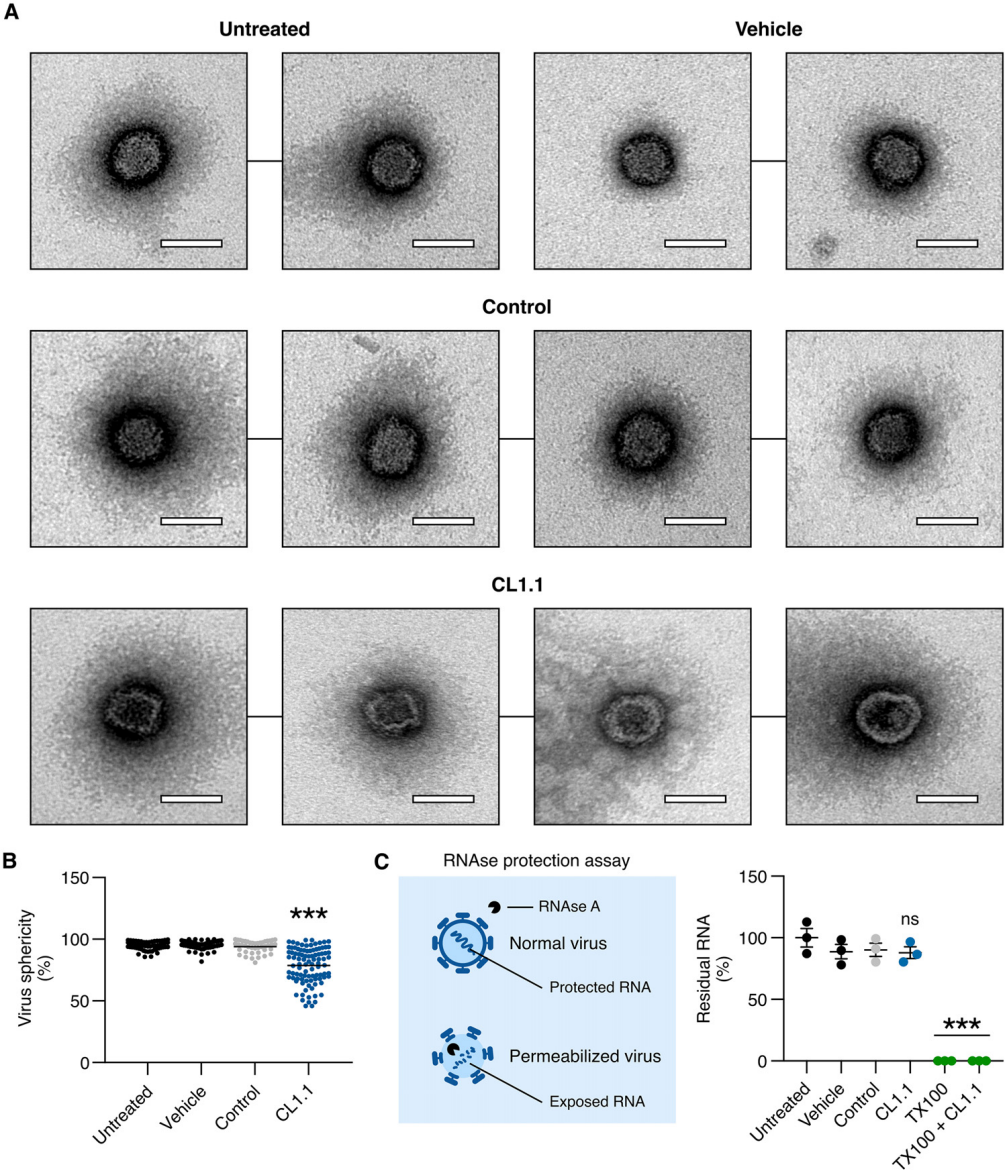
Effect of CL1.1 on ZIKV ultrastructure and envelope integrity. (A) ZIKV (6.5 × 10^10^ PFU/mL, H/PF/2013 strain) was treated with 10 μM CL1.1 for 1 h. The viruses were then analyzed by electron microscopy (the control peptide was CL7.4, and the vehicle was DMSO). Fields with individual virions are presented. Scale, 50 nm. (B) The sphericity of viral particles was assessed by calculating the ratio between the height (*h*) and the length (*l*) of the particles. Spherical particles have an *h*:*l* ratio of 1, and elongated particles have an *h*:*l* ratio smaller than 1. (C) ZIKV integrity was assessed by an RNase A protection assay. ZIKV (Brazil/2016/INMI1 strain) was treated with 10 μM CL1.1 or 1% Triton X-100 (with and without CL1.1) for 1 h, followed by treatment with RNase A for 1 h. Residual viral RNA was quantified by RT-qPCR (compared to control peptide CL7.4 or vehicle alone). Data are presented as individual biological replicates and mean ± SEM; ***, *P ≤ *0.001; ns, *P* > 0.05 ((B) Kruskal-Wallis ANOVA followed by Dunn’s *post hoc*; (C) one-way ANOVA followed by Dunnett’s *post hoc*). Uncropped source pictures are available in Fig. S8 in Supplemental File 1.

We treated a suspension of ZIKV from the Asian lineage (6.5 × 10^10^ PFU/mL) with 10 μM CL1.1 or a control peptide for 1 h. The viruses were then fixed and analyzed in negative staining electron microscopy. Under control conditions, all free viral particles were well defined (Fig. 5A) and spherical (Fig. 5B). In contrast, in the presence of CL1.1, the particles were angular (Fig. 5A) and significantly less spherical (Fig. 5B). The perimeter of the CL1.1-treated viruses was also noticeably less electron-dense, whereas the core of the viruses remained electron-dense (Fig. 5A).

Such changes in ultrastructure could lead to loss of viral integrity. We tested whether CL1.1 treatment could disorganize the capsid and lead to the release of viral RNA or make it accessible to RNase A. Using an RNase protection assay (30), we found that the amount of viral RNA was identical to the untreated virus after treatment with 10 μM CL1.1 or in control peptide, indicating that the genome was well protected in the presence of the peptide. As a control, we used 1% Triton X-100, a detergent commonly used to disrupt lipid envelopes. As expected, viral RNA was degraded upon RNase A treatment in the presence of Triton X-100 (Fig. 5C). Of note, CL1.1 had no inhibitory effect on the RNase A activity because combining Triton X-100 and CL1.1 yielded the same result as the treatment with Triton X-100 alone (Fig. 5C).

These data indicated that CL1.1 induced significant changes in the structure of ZIKV particles but did not render viral RNA accessible. We speculated that the peptide bound to the lipid envelope of the virus through its hydrophobic face, rigidifying the envelope, which induced the morphology changes.

### CL1.1 and CL7.1 may also interact with the ZIKV E protein.

The amphipathicity of the peptides seems important for ZIKV inhibition. However, the control peptide CL7.4, which displayed a similar amphipathic of μH = 0.28, exhibited no antiviral activity. We hypothesized that the antiviral peptides may also interact with the viral E protein.

**FIG 6:**
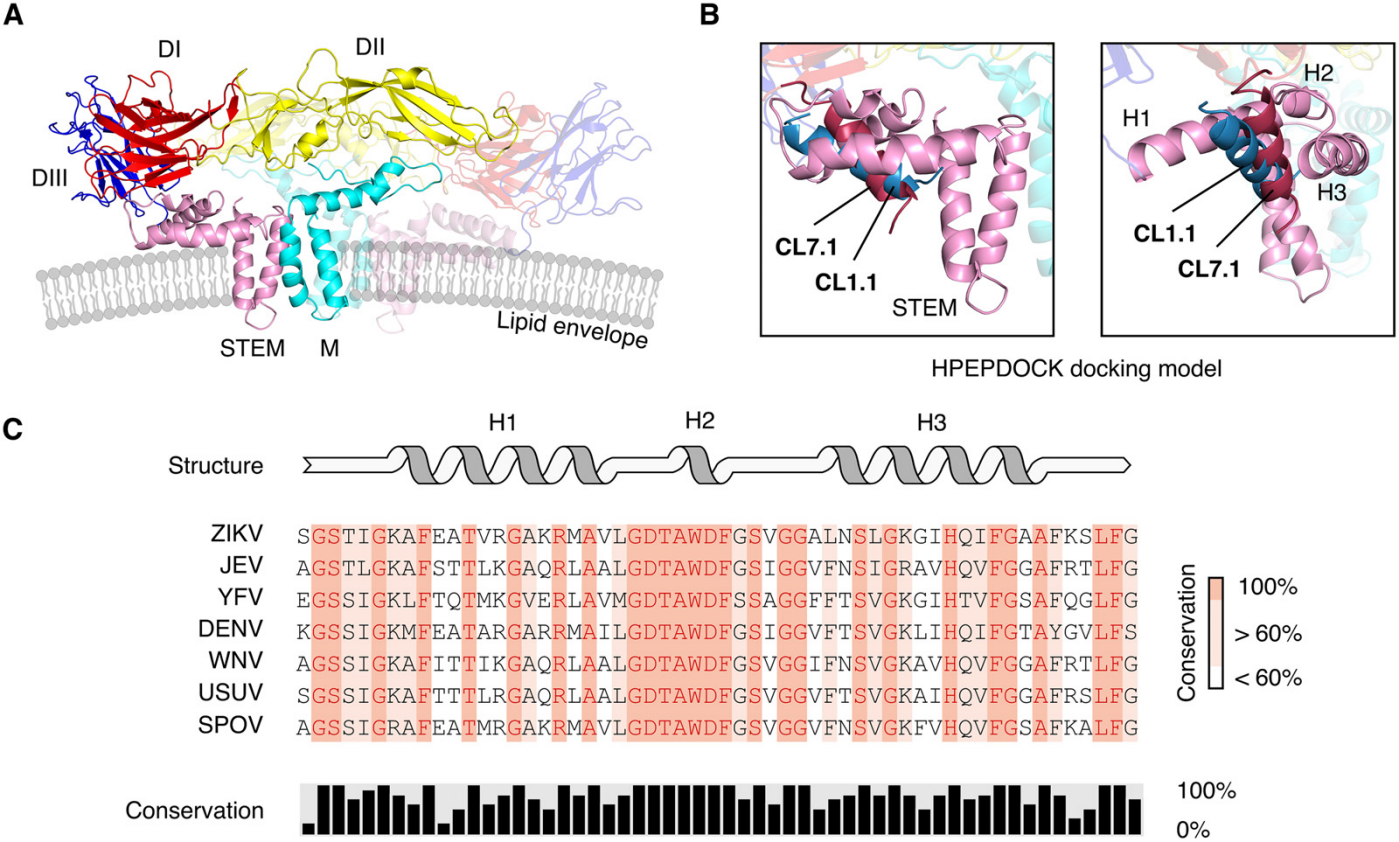
Claudin-derived peptides docking to ZIKV surface proteins. (A) The surface of the ZIKV virion was decorated by membrane (M) and envelope (E) proteins that form a dimer. The different domains of the E protein (STEM, DI to DIII) are indicated (in pink, red, yellow, and blue). The lipid envelope of the virus is shown. PDB accession number 5IRE. (B) Molecular docking prediction of CL7.1 (red) and CL1.1 (blue) to the ZIKV surface proteins was obtained using HPEPDOCK. CL1.1 and CL7.1 insert in-between membrane-proximal alpha helixes H1 and H3 of the stem domain of E (pink). The right panel is a 70-degree rotation along the *y*-axis of the left panel. (C) Sequences alignment of stem domains of E proteins from ZIKV, JEV, YFV, DENV, WNV, USUV, and SPOV is presented. The percentage amino acid conservation between the viruses and the corresponding secondary structure are indicated. DI to DIII, domains I to III of the envelope protein; STEM, stem domain of the envelope protein; H1 to H3, alpha helixes of the stem domain; M, membrane protein.

We used HPEPDOCK, a protein-peptide docking software (31), to identify the putative interactions between the claudin-derived peptides and the ZIKV E protein in its prefusion state (32) because the interaction likely occurred before the pH-dependent conformational change (Fig. 6A). The software predicted that CL1.1 and CL7.1 would bind to the stem region between the two envelope-proximal alpha helices H1 and H3 (Fig. 6B). The hydrophilic faces of the peptides would participate in the protein-peptide interactions and the hydrophobic faces would be in close contact the lipid envelope (Fig. 6B). No such interaction was predicted for the control peptide (Fig. S5 in Supplemental File 1).

These results suggested that the peptides may bridge the stem domain of the ZIKV E protein near the viral envelope.

### CL1.1 neutralized other flaviviruses but not unrelated enveloped viruses.

Our data suggested that the inhibitory peptides may act through interaction with both the lipid envelope of the virion and the ZIKV E proteins. Alignment of the sequence of the ZIKV stem domain with the corresponding sequences of other flaviviruses revealed that the region was highly conserved among flaviviruses (Fig. 6C). Therefore, we postulated that the peptides would exhibit a broad anti-flavivirus activity. We tested the activity of CL1.1 (the most effective peptide) on JEV and YFV infections.

**FIG 7:**
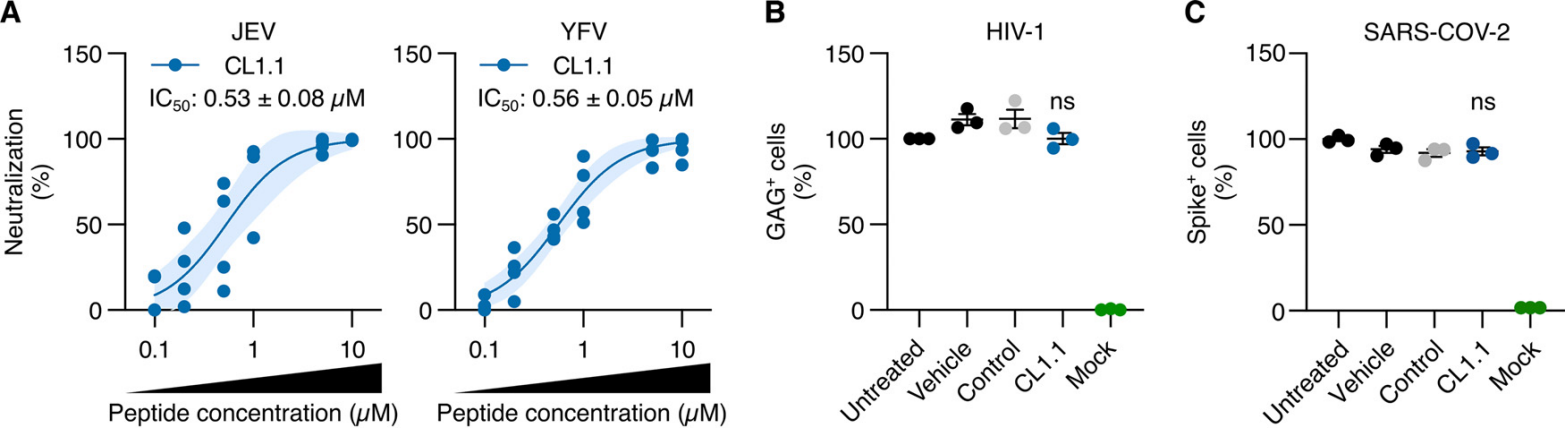
CL1.1 neutralized other flaviviruses. (A) Dose-dependent antiviral activity of the CL1.1 was tested on the infection Vero E6 with JEV (MOI of 1, JEV/G3/RP-9 strain) and YFV (MOI of 1, YFV/17D strain). Viral production was assessed by titrating the supernatants. The dark line represents the fitted dose-response model, and the light area represents the 95% confidence interval of the model. The calculated IC_50_ is indicated. (B) HIV-1 (50 ng/mL) was treated with 50 μM CL1.1 for 1 h. This inoculum was used to infect primary CD4^+^ T lymphocytes for 3 h. After infection, the unbound virus was washed and cells were kept in a fresh medium containing peptides for 72 h. Viral infection was assessed through flow cytometry on Gag^+^ cells (compared to untreated cells; vehicle corresponds to DMSO, control peptide was CL7.4). (C) SARS-CoV-2 (MOI of 1) was treated with 50 μM CL1.1 for 1 h. This inoculum was adsorbed on Vero E6 cells for 2 h. Then, the unbound virus was washed away, and cells were kept in a fresh medium containing peptides for 72 h. Viral infection was assessed by flow cytometry on Spike^+^ cells (compared to untreated cells; vehicle corresponds to DMSO, control peptide to CL7.4). Data are presented as individual biological replicates and mean ± SEM; ns, *P* > 0.05 (one-way ANOVA followed by Dunnett’s *post hoc*).

JEV and YFV inocula (5 × 10^5^ PFU/mL) were treated with various concentrations of CL1.1, and we tested the efficacy of infection of Vero E6 cells (MOI of 1) as determined by titration. We found that CL1.1 effectively inhibited the infection by JEV and YFV in Vero E6 cells in a dose-dependent manner. The IC_50_ was comparable to that of ZIKV with values of 0.87 ± 0.11 μM, 0.53 ± 0.08 μM, and 0.56 ± 0.05 μM for ZIKV, JEV, and YFV, respectively (Fig. 1C and 7A). In line with our previous observations, the antiviral properties of d-CL1.1 were comparable to that of CL1.1 (Fig. S6 in Supplemental File 1).

We finally wondered whether the claudin-derived peptides could exhibit a broader antiviral function, and tested the antiviral activity of 50 μM CL1.1 against HIV-1 and SARS-CoV-2, two unrelated enveloped viruses. No antiviral activity of CL1.1 against either HIV-1 infection of human primary CD4^+^ T cells or SARS-CoV-2 infection of Vero E6 cells was detected using flow cytometry (Fig. 7B to C).

In conclusion, CL1.1 seemed to display a specific anti-pan-flavivirus activity.

## DISCUSSION

We identified two helical peptides derived from the N-terminal sequences of human claudin-1 and claudin-7 (named CL1.1 and CL7.1, respectively) that exhibit anti-ZIKV properties. Fusion assays on mosquito cells revealed that the peptides blocked the pH-dependent E-mediated fusion. CL1.1 was more potent than CL7.1. We also found that CL1.1 exhibited broad-spectrum anti-flavivirus properties, as it inhibited infection of Vero E6 cells by JEV and YFV. Inhibition was independent of the target cell type and antiviral peptides appeared to interact directly with the ZIKV particles. Claudin-derived peptides were predicted to form amphipathic alpha helices. These amphipathic helices likely interact with the virus lipid envelope. Electron microscopy observations revealed that treatment of virions with CL1.1 modified the shape of the virions. *In silico* prediction also suggested that claudin-derived peptides may engage with the stem domain of ZIKV E protein. We, therefore, propose that claudin-derived peptides inhibit the E-mediated fusion by simultaneously interacting with the virus lipid envelope and the E-protein, preventing E-protein conformational changes or alterations in lipid curvature, which are both necessary for the fusion.

We recently demonstrated that claudin-7 is required for optimal ZIKV replication in human endothelial cells (15). We initially expected that the claudin-7-derived peptides spanning over the putative ZIKV binding site would bind to the E protein and prevent the interaction of the virus with claudin-7. Such a strategy was used in other settings (33). Intriguingly, none of the peptides corresponding to extracellular domains of claudin-7 had a significant impact on ZIKV infection of hCMEC/D3 cells. The lack of viral interference with the infection could be the consequence of a poor conformation of the peptides. Extracellular domains of claudins were composed of beta sheets (Fig. S1 in Supplemental File 1) while the peptides likely form alpha helices in solution (Fig. 4A).

Nevertheless, we found that the peptide corresponding to the N-terminal 18-mer of claudin-7, named CL7.1, inhibits ZIKV infection in five human cell lines. This was reminiscent of a previous study demonstrating that the corresponding peptide derived from claudin-1, named here CL1.1, inhibits HCV infection in hepatoma Huh7.5.1 cells (19). CL1.1 is more efficient than CL7.1 in blocking ZIKV infection. Of note, CL1.1 and CL7.1 only have 4 amino acid differences, but their antiviral efficacy differs by almost 1 log, evidencing the high specificity of these peptides.

The efficacy of CL1.1 was intriguing since claudin-1 is not expressed in hCMEC/D3 cells (18). Similarly, we found that CL7.1-mediated inhibition is comparable between endothelial and epithelial cells but previously found that claudin-7 does not seem to play a role in epithelial cell infection (15). We concluded that inhibition was not achieved by interfering in the interaction between prM/E and claudin. This led us to search for a mechanism of inhibition that is independent of expression levels of claudin expression in cells.

When the treatment of ZIKV particles with the claudin-derived peptides was followed by dilution and culture with the cells, we found that the inhibition was retained although slightly lower than that obtained by retaining peptides in the culture medium throughout the experiment. This could be the result of reversibility in the virus-peptide interactions.

The reversibility of the interaction between CL1.1 and the HCV E1/E2 proteins has previously been demonstrated by ultracentrifugation (19). Differences in inhibition efficacy could also be explained by the fact that peptides present in the culture medium could bind and inhibit newly formed particles.

Many antiviral peptides have been described over the years, including against flaviviruses, and their mechanisms of action are diverse. Several models suggest that antiviral peptides could induce particle aggregation and/or prevent viral entry to the cell (10, 34, 35). Here, CL1.1 did not seem to alter viral binding to the cell membrane or internalization. Electron microscopic observations did not evidence particle aggregation. In other models, antiviral peptides are virucidal as they form pores in the viral lipid envelope, disrupting viral integrity (7–10, 30). We found no such effect. Viral RNA was still protected from RNase in particles upon claudin-derived peptide treatment. Finally, some antiviral peptides have been shown to block viral fusion (36), which seems to be the case for the claudin-derived peptides.

Our findings highlighted the importance of the amphipathicity of CL1.1 and CL7.1 in their ability to inhibit ZIKV infection. Amphipathicity has been previously reported as key for the antiviral activities of peptides (37). Both l- and d-enantiomers of CL1.1 showed an identical affinity for ZIKV, YFV, and JEV. It is recognized that chiral peptides retain their hydrophobic properties but should have altered affinities for proteins (38). Since the control peptide CL7.4 has a similar amphipathicity as CL1.1, it suggests that CL1.1 antiviral activity does not depend solely on peptide-lipid interactions, but that some interaction between the peptide and the E protein may also be at play.

*In silico* analyses suggested that CL1.1 and CL7.1 may directly interact with the stem domain of the ZIKV E protein in an envelope-proximal pocket. This is consistent with the previous study that showed direct binding of HCV E1/E2 to CL1.1 (19). The stem domain is highly conserved among flaviviruses, and we found that CL1.1 was also able to block both JEV and YFV infections. Other groups also suggested that peptides derived from, or interacting with, the stems of other flaviviruses displayed broad-spectrum antiviral properties (8, 36, 39). The stem domain seemed to constitute a promising target for anti-flavivirus drug design.

The amphipathicity of the peptides combined with the putative location of the interaction with the E protein (*in silico*) suggest that claudin-derived peptides likely bridge the E protein with the virus lipid envelope. This will lead to envelop rigidification, hence the change in sphericity observed by electron microscopy. The lack of antiviral activity of CL1.1 against HIV-1 or SARS-CoV-2 infections could then be related to differences in the lipid composition of viral envelopes along with a lack of interaction sites with Env and spike proteins.

Peptide-based treatments present several advantages. These molecules are often not cytotoxic, easy to manufacture, and have a high affinity to their targets (6). In addition, peptides can be designed to penetrate tissues and thus gain access to organs normally protected by barriers, such as the blood-brain barrier (9) or the blood-placental barrier (8, 40), both of which are of interest in preventing severe ZIKV brain infections in adults and fetuses. In some cases, claudin-derived peptides have been described as potent disruptors of tight junctions *in vitro* and *in vivo*, which could promote virus dissemination into some tissues and could lead to impaired organ functions (41–44). Our results showed that CL1.1 did not alter epithelial monolayer integrity.

The main limitation to the use of peptides in therapy is their limited persistence *in vivo*, mainly due to degradation by proteases. To counteract this phenomenon, d-enantiomers could be a good alternative, because they have the same efficacy *in vitro* and are less susceptible to degradation *in vivo* (28, 45).

Our findings provided a basis for the development of compounds that could target a large range of the known flaviviruses (e.g., ZIKV, JEV, YFV) and could also be efficient against flaviviruses that may emerge in the future. The development of such a molecule would be central to the preparation for future epidemics.

## MATERIALS AND METHODS

### Cells.

Human embryonic kidney HEK 293T (ATCC, CRL-11268), human lung A549 (ATCC, CCL-185), human colon Caco-2/TC7 (Merck, SCC209), and African green monkey kidney Vero E6 (ATCC, CRL-1586) epithelial cell lines were maintained in high-glutamine and high-glucose DMEM (Thermo Fisher) supplemented with 10% heat-inactivated fetal bovine serum (FBS; Thermo Fisher), 100 U/mL penicillin and 100 μg/mL streptomycin (Thermo Fisher).

Aedes albopictus embryonic C6/36 (ATCC, CRL-1660) cells were maintained in Leibovitz’s L-15 medium (Thermo Fisher) supplemented with 10% FBS and antibiotics.

Human cerebral microvascular hCMEC/D3 (Merck, SCC066), human bone marrow TrHBMEC (46), and primary human umbilical vein HUVEC (ATCC, CRL-1730) endothelial cells were maintained in complete EndoGRO Basal Medium (Merck) supplemented with 5% FBS, 0.2% EndoGRO-LS supplement, 5 ng/mL rhEGF, 10 mM l-glutamine, 1 μg/mL hydrocortisone hemisuccinate, 0.75 U/mL heparan sulfate, 50 μg/mL ascorbic acid (Merck), and antibiotics.

Human primary CD4^+^ T lymphocytes were isolated from the blood of healthy donors provided by the EFS (Etablissement Français du Sang, the official French blood bank) as previously described (47). The cells were activated with 1 μg/mL phytohaemagglutinin (Oxoid) for 24 h and then cultivated in RPMI supplemented with 10% FBS, 50 IU/mL human IL-2, and antibiotics. Primary CD4^+^ T lymphocytes were left to expand for 5 to 7 days before use.

All cells were kept at 37°C with 5% CO_2_ except for C6/36 which were kept at 28°C with no CO_2_.

### Viruses.

Zika viruses (ZIKV) were produced on Vero E6 cells. Experiments were performed on strain AF/1991/HD78788 (African lineage; GenBank accession no. KF383039) unless otherwise indicated. Other ZIKV strains were tested, including Brazil/2016/INMI1 (Asian lineage, GenBank accession no. KU991811) and H/PF/2013 (Asian lineage; GenBank accession no. KY766069). Japanese encephalitis virus strain JEV/G3/RP-9 (48) and yellow fever vaccine strain YFV/17D (GenBank accession no. MG051217) were produced on C6/36 cells.

HIV NL4-3 strain was produced by calcium-phosphate transfection of HEK-293T cells as previously described (47).

The SARS-CoV-2 isolate BetaCoV/France/IDF00372/2020 (EVAg collection, Ref-SKU no. 014V-03890) was kindly provided by the National Reference Centre for Respiratory Viruses hosted by Institut Pasteur and headed by S. van der Werf.

All infections were performed in 2% FBS media in biosafety level (BSL)-3 facilities.

### Peptides.

Claudin-derived peptides used in this study (of which sequences are detailed in Table S1 in Supplemental File 1) were synthesized by GenScript (Netherlands). The peptides were initially dissolved at a concentration of 5 mM in DMSO (Merck) as stock solution and were then used at various concentrations from stock as indicated. The final concentration of DMSO was never greater than 1% (vol/vol) except in the cytotoxicity assay.

As controls for our experiments, we intended to use scrambled versions of CL7.1 and CL1.1. After multiple failed attempts to synthesize these controls, we opted to use CL7.4, a peptide from our screen that did not have a significant effect on ZIKV infection but shared common features to CL7.1 and CL1.1 (i.e., similar alpha-helical structure and amphipathicity value μH = 0.28, see Fig. S4 in Supplemental File 1).

### Viral replication assay.

Infected cells were washed with PBS and intracellular RNA was extracted using the Nucleospin 96 RNA kit (Macherey-Nagel). One-step RT-qPCR was performed using the GoTaq 1-Step RT-qPCR kit (Promega) with the following protocol: 15 min at 37°C, 10 min at 95°C followed by 40 cycles consisting of 10 s at 95°C, 30 s at 60°C, and 30 sec at 72°C (QuantStudio 6 Flex real-time PCR system, Applied Biosystems). The melting curve was obtained to certify specific amplification. Genome equivalent concentration was assessed with the standard curve method using known concentrations of synthetic RNA fragments corresponding to the ZIKV *NS5* coding region and was normalized to the *GAPDH* level.

The ZIKV *NS5* primers used were 5′-AAGTACACATACCAAAACAAAGTG (forward) and 5′-TCCGCTCCCCCTTTGGTCTTG (reverse). The *GAPDH* primers used were 5′-GGAGCGAGATCCCTCCAAAAT (forward) and 5′-GGCTGTTGTCATACTTCTCATGG (reverse).

### Viral titration assay.

Supernatants of ZIKV and YFV infected cells were sequentially diluted 10-fold and inoculated onto Vero E6 monolayers in 24-well plates. After 1 h of incubation, 2% carboxymethyl cellulose (CMC; Merck) overlay containing 2% FBS culture medium was added to each well. The cells were then incubated at 37°C for 5 days. Following the incubation, the CMC overlay was removed and a PBS – paraformaldehyde 4% (Electron Microscopy Sciences) solution was added to each well and incubated at room temperature for 15 min. The fixation solution was removed, and plates were washed three times with PBS. Plates were then stained with a 1% crystal violet solution (Merck). The titer was expressed as PFU.

Alternatively, for JEV infected cells, plates were incubated at 37°C for 2 days after adding the CMC overlay. They were then washed and fixed as described earlier. Cells were then permeabilized with PBS-Triton 0.2% for 3 min, washed three times, and incubated in blocking buffer (PBS, Tween 20 0.1%, and FBS 1%) for 30 min followed by 2 h of incubation with pan-Flavivirus antibody 4G2 purified from the ATCC hybridoma. Plates were washed three times with PBS followed by an hour-long incubation with a secondary antibody conjugated to horseradish peroxidase (Bio-Rad). Detection was achieved using the VIP Peroxidase Assay (Vector) according to the manufacturer’s recommendations. The titer was expressed as foci-forming units (FFU).

### Cytotoxicity assay.

Confluent A549 monolayers were treated with 0 to 200 μM CL1.1 or control peptide for 48 h at 37°C. We used an equivalent volume of DMSO as a control. Following the treatment, the medium was swapped for fresh DMEM with 2% FBS in the presence of 1.2 mM MTT. Mitochondrial reduction of MTT into Formazan was allowed for 4 h. Formazan was dissolved in isopropanol-HCl and absorbance at 570 nm was measured with a Multiskan FC Microplate Photometer (Thermo Fisher).

### Tight-junctions disruption assay.

Caco-2/TC7 cells were cultured on Transwell inserts (12 mm diameter, 3 μm porosity, polycarbonate, Costar) for 21 days, delimiting apical (upper chamber) and basolateral (lower chamber) compartments. Apical and basolateral media were replaced every 2 days, and transepithelial electric resistance (TEER) was routinely analyzed (EVOM World Precise Instruments). Once fully differentiated, the monolayers were treated with 50 μM CL1.1 or control peptide and TEER was measured at different time points over a 24 h period. We used 10 μM EDTA (Thermo Fisher) as a control.

### Binding and internalization assay.

Cells were seeded in 2% FBS medium 24 h before infection. Prechilled cells were incubated with ZIKV (MOI of 10) for 1 h at 4°C – to block the internalization of the viruses – in the presence of 10 μM CL1.1 or control peptide. We used 200 μg/mL Heparin (Merck) as a control. The cells were then washed three times with cold PBS to remove unbound particles. Cell surface adsorbed ZIKV particles were analyzed by harvesting RNA at this step.

ZIKV internalization was induced by adding a warm medium and keeping the cells at 37°C for 2 h in the presence of 10 μM CL1.1 or a control peptide. We used 100 μM Dynasore (Merck) as a control. The cells were then treated with 0.05% Trypsin (Thermo Fisher) to remove uninternalized particles, cells were pelleted, and RNA was harvested.

### Cell-to-cell fusion assay.

Confluent C6/36 monolayers were infected with ZIKV (MOI of 2) for 48 h to ensure all the cells were infected before the experiment. The cells were then incubated in fresh RPMI 1640 medium (Thermo Fisher) incubated for 1 h at 37°C in the presence of 10 μM CL1.1, or 50 μM CL7.1, or 50 μM the control peptide (i.e., CL7.4). We used 500 ng/mL 4G2 MAb (ATCC hybridoma) as a control. The medium was then swapped for RPMI-10 mM MES (pH 5.5) or RPMI-HEPES 10 mM (pH 7.0) and cell-to-cell fusion was allowed for 2 h at 37°C. Cells were then fixed with isopropanol for 15 min at −20°C, air-dried, and stained with 10% Giemsa solution (RAL Diagnostics). Cell-to-cell fusion was visualized with bright field microscopy (EVOS M5000, Thermo Fisher). Fusion-index (FI) was quantified using the following formula: 1 – (number of cytoplasms/number of nuclei). For the quantification, fields with 200 ± 50 nuclei were considered.

### *In silico* peptide structure predictions.

Structures of the peptides were predicted using the PEP-FOLD3 software (25) hosted on the Mobyle platform of the French Ressource Parisienne en Bioinformatique Structurale (49). The predictions were made using the sequences of CL1.1 and CL7.1, 100 simulations were performed, and the models were ranked by sOPEP, the best model was considered for analysis.

Amphipathicity of the peptides was explored using HELIQUEST (26). Calculations for the amphipathicity value (μH) of the peptides were conducted using the Eisenberg scale (27).

### Electron microscopy.

ZIKV was first concentrated by polyethylene glycol 6000 (Merck) precipitation and purified by centrifugation in a discontinued gradient of sucrose. These sucrose-purified viruses were used for the electron microscopy experiments.

A ZIKV stock (5 × 10^8^ PFU) was mixed with 10 μM CL1.1 or control peptide for 1 h at 37°C. The different mixtures were then fixed by the addition of 4% PFA and 1% glutaraldehyde in 0.1 M phosphate buffer (pH 7.2) before being placed in contact with Formvar/carbon-coated nickel grids for 5 min. Negative staining was performed using 2% uranyl acetate (Agar Scientific) followed by transmission electron microscope analyses (JEM 1011, JEOL).

### RNase A protection assay.

A ZIKV stock (1 × 10^6^ PFU, H/PF/2013 strain) was mixed with 10 μM CL1.1 or control peptide for 1 h at 37°C. We used 1% Triton X-100 (Merck) as a control. Then, RNase A (Thermo Fisher) was added to a final concentration of 100 μg/mL and incubated for 1 h at 37°C. Ribolock RNase inhibitor (Thermo Fisher) was added to a final concentration of 400 U/mL and incubated for 10 min at 37°C. Viral RNA was harvested from the solution using the QIAamp Viral RNA kit (Qiagen) and RT-qPCR was performed as described above. To ensure that CL1.1 does not prevent RNase A degradation, we used a condition with Triton X-100 mixed with RNase A.

### *In silico* peptide-protein interaction predictions.

The interaction model of CL1.1 and ZIKV envelope protein (PBD accession code 5IRE) was generated using HPEPDOCK (31) using the CL1.1 structure predicted earlier with PEP-FOLD3.

### Flavivirus stem sequences alignment.

Sequences of the stem domains of the E protein from ZIKV (GenBank accession no. AHZ13508), JEV (GenBank accession no. M55506), YFV (GenBank accession no. AAC35899), DENV (GenBank accession no. AAB70694), WNV (GenBank accession no. AAA48498), Usutu virus (USUV; GenBank accession no. AAS59402) and Spondweni virus (SPOV; GenBank accession no. AOZ57820) were aligned using MUSCLE.

### Flow cytometry.

For Gag staining, infected cells were fixed in 4% paraformaldehyde (PFA) for 10 min and incubated with the KC57-PE monoclonal antibody (Beckman Coulter) in PBS, 1% BSA, and 0.05% Saponin (Merck) for 30 min at 4°C. Samples were analyzed on a FACSCanto II (BD Biosciences).

For Spike staining, infected cells were fixed in 4% PFA for 10 min and staining was performed in PBS, 1% BSA, 0.05% sodium azide, and 0.05% Saponin. Cells were stained with the primary pan-SARS-CoV-2 anti-S mAb102 human monoclonal antibody (50) and secondary Alexa Fluor 477 (Thermo Fisher) for 30 min at 4°C. Samples were analyzed on an Attune NxT (Thermo Fisher).

### Quantification and statistical analyses.

Graphical representations and statistical analyses were performed using GraphPad Prism version 8.2 for macOS (GraphPad Software). Following a positive normality test, data were analyzed with a one-way analysis of variance (ANOVA) followed by Dunnett’s *post hoc*. Otherwise, data were analyzed with a nonparametric Kruskal-Wallis ANOVA followed by Dunn’s *post hoc*.
