# Supplementary material for: Human Claudin-Derived Peptides Block the Membrane Fusion Process of Zika Virus and Are Broad Flavivirus Inhibitors
Source: Microbiol Spectr. 2022 Aug 30;10(5):e02989-22. doi: 10.1128/spectrum.02989-22 (PMC9603178; doi:10.1128/spectrum.02989-22)
Supplement: Supplemental file 1 — Supplemental material. Download spectrum.02989-22-s0001.pdf, PDF file, 0.7 MB [file spectrum.02989-22-s0001.pdf]

Name	Sequence	Remarks
CL1.1	MAN**A**GLQLLGF**IL**A**F**LGW	
CL7.1	MAN**S**GLQLLGF**SM**A**L**LGW	
CL7.3	IPQWQMSSYAGDNIITAQ	
CL7.4	IITAQAMYKGLWMDCVTQ	Used as control peptide
CL7.6	MYDSVLALSAALQATRAL	
CL7.9	TRCGGDDKVKKARIAMGG	
CL7.10	IAMGGGIIFIVAGLAALV	
CL7.12	IVTDFYNPLIPTNIKYEF	
CL7.16	RVPRSYPKSNSSKEYV	
CL1.1–F	MANAGLQLLGFILALLGW	
CL7.1+F	MANSGLQLLGFSMAFLGW	
CL1.1+2S	MANSGLQLLGFSLAFLGW	
CL7.1–2S	MANAGLQLLGFIMALLGW	
CL7.2	ALLGWVGLVACTAIPQWQ	
CL7.5	DCVTQSTGMMSCKMYDSV	
CL7.7	ATRALMVVSLVLGFLAMF	
CL7.8	FLAMFVATMGMKCTRCGG	
CL7.11	LAALVACSWYGHQIVTDF	Synthesis failure*
CL7.13	IKYEFGPAIFIGWAGSAL	
CL7.14	AGSALVILGGALLSCSCP	
CL7.15	SCSCPGNESKAGYRVPRS	

S1 Table. Sequences of the peptides described in this study.

*These peptides could not be synthesized because they were too hydrophobic and aggregated.

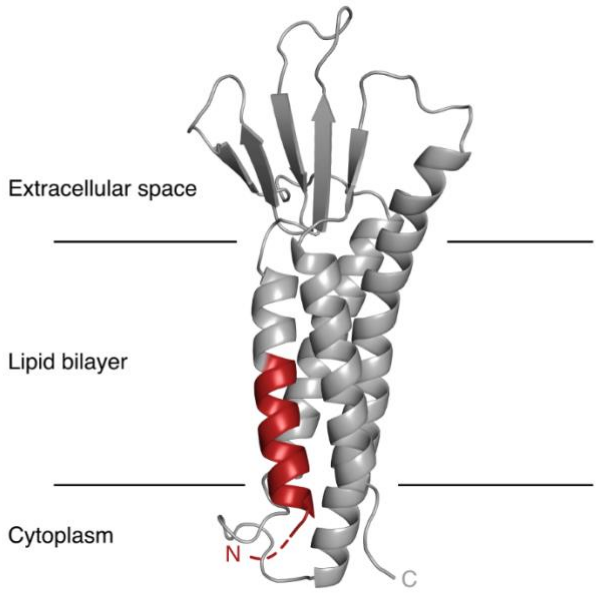

S1 Fig. Position of CL7.1 on parental claudin-7.

Claudin-7 sequence was modeled on the structure of claudin-9 (PBD accession code: 6OV2). CL7.1 sequence is highlighted on the structure.

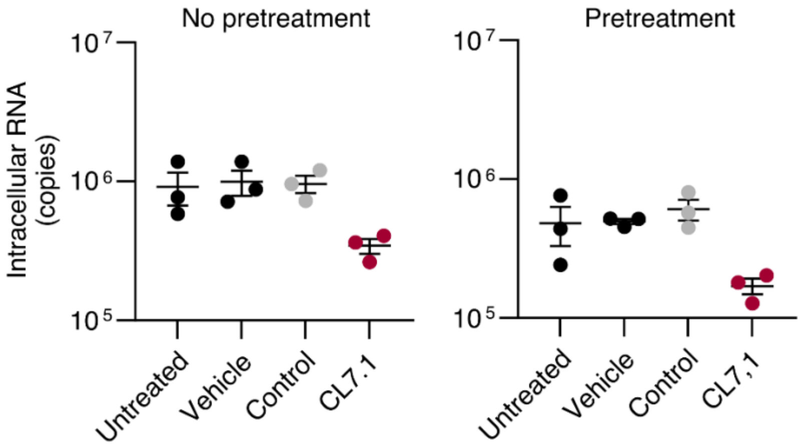

S2 Fig. Peptide treatment prior to infection is not required for inhibition.

ZIKV inoculum (5×10^5 PFU/mL, equivalent to a MOI of 1) was suspended in medium containing 50 µM of CL7.1 or control peptide (CL7.4). These inocula were used to infect hCMEC/D3 cells for 2 h directly after suspension (left panel) or following a 1 h treatment in the presence of peptide (right panel). The cells were subsequently washed and kept in fresh medium containing peptides for 48 h. Viral replication was assessed by RT-qPCR.

Data information: Data are presented as individual biological replicates.

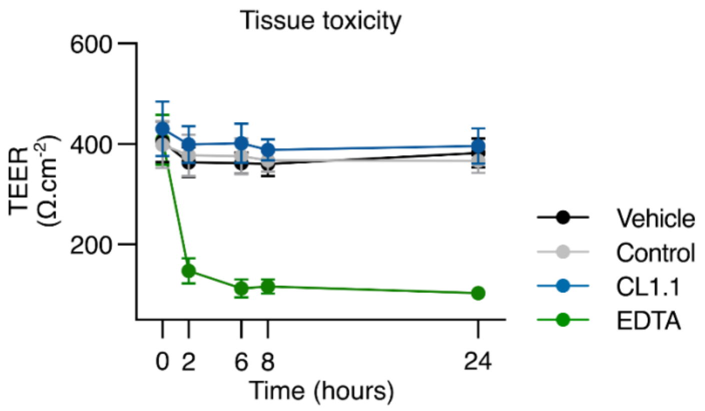

S3 Fig. CL1.1 treatment does not alter tight junctions.

Caco-2 monolayers were differentiated into an intestinal barrier on transwell devices. Monolayers were treated with 50 µM of CL1.1, or control CL7.4, or DMSO (vehicle), or 10 µM EDTA (positive control). Tissue integrity was monitored by measuring the trans-epithelial electric resistance for 24.

Data information: Data are presented as mean ± SEM (n = 3 biological replicates).

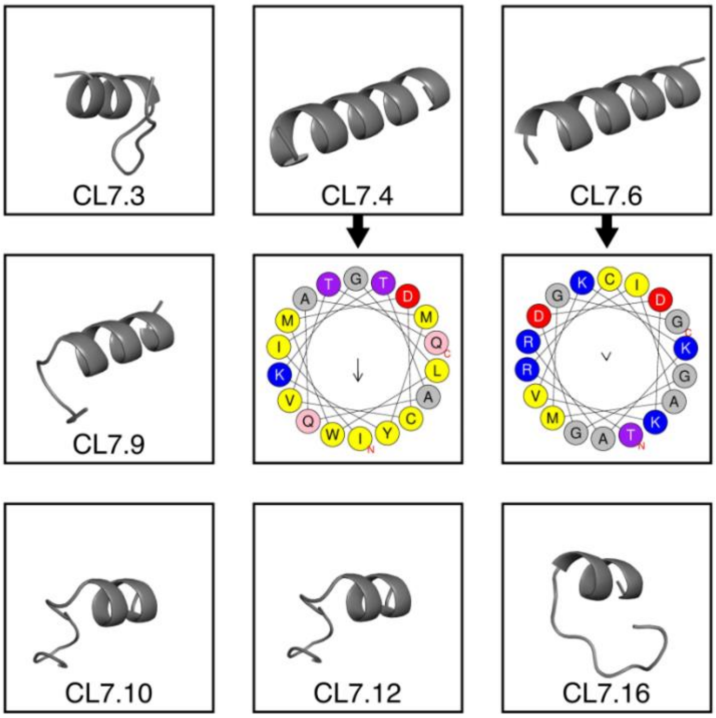

S4 Fig. Structure and properties of inactive claudin-7-derived peptides.

The tridimensional structures of CL7.3, CL7.4, CL7.6, CL7.9, CL7.10, CL7.12 and CL7.16 were predicted using PEP-FOLD3. Peptides that formed continuous alpha helixes were projected on helical wheels. Amphipathicity values (μH) for CL7.4 (0.282) and CL7.6 (0.019) were calculated with HELIQUEST.

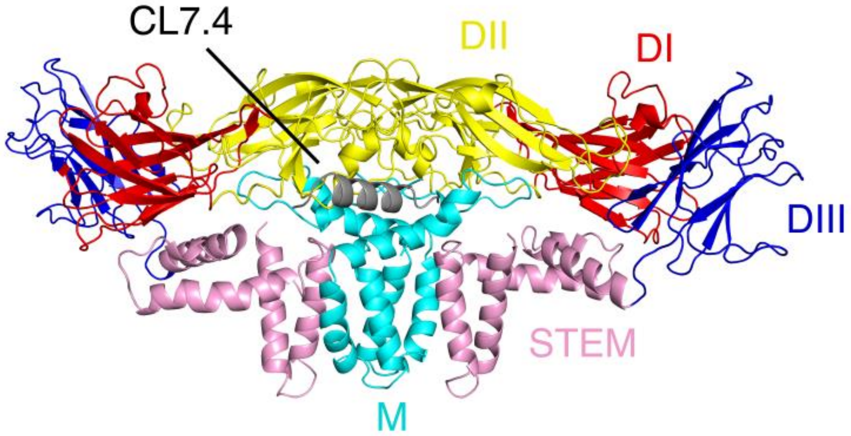

S5 Fig. CL7.4 docking to ZIKV surface proteins.

Molecular docking of CL7.4 to the ZIKV surface proteins using HPEPDOCK.

Abbreviations: DI to DIII: domains I to III of the envelope protein; STEM: stem domain of the envelope protein; H1 to H3: alpha helixes of the stem domain; M: membrane protein.

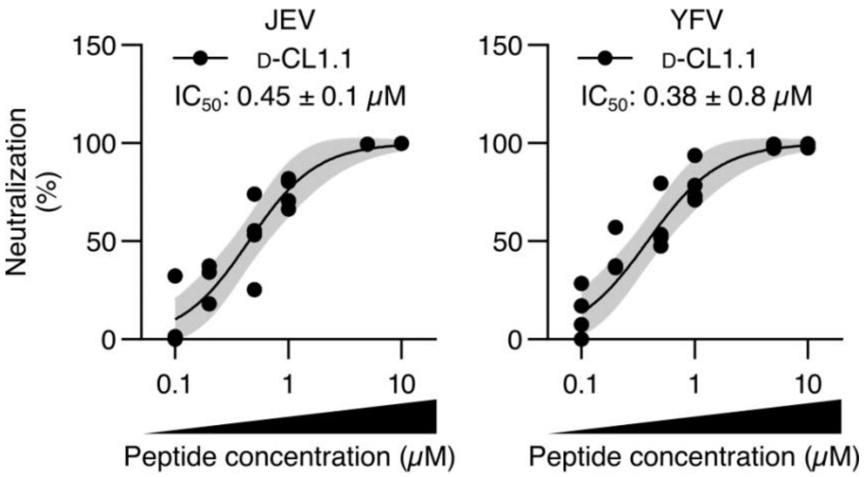

S6 Fig. D-CL1.1 neutralizes other flaviviruses.

Increasing concentrations of the D-CL1.1 were tested on Vero E6 cells against JEV (MOI of 1) and YFV (MOI of 1). Viral production was assessed by titrating the supernatants. The dark line represents the fitted dose-response model, and the light area represents the 95% confidence interval of the model. The calculated IC50 are indicated.

Data information: Data are presented as individual biological replicates.

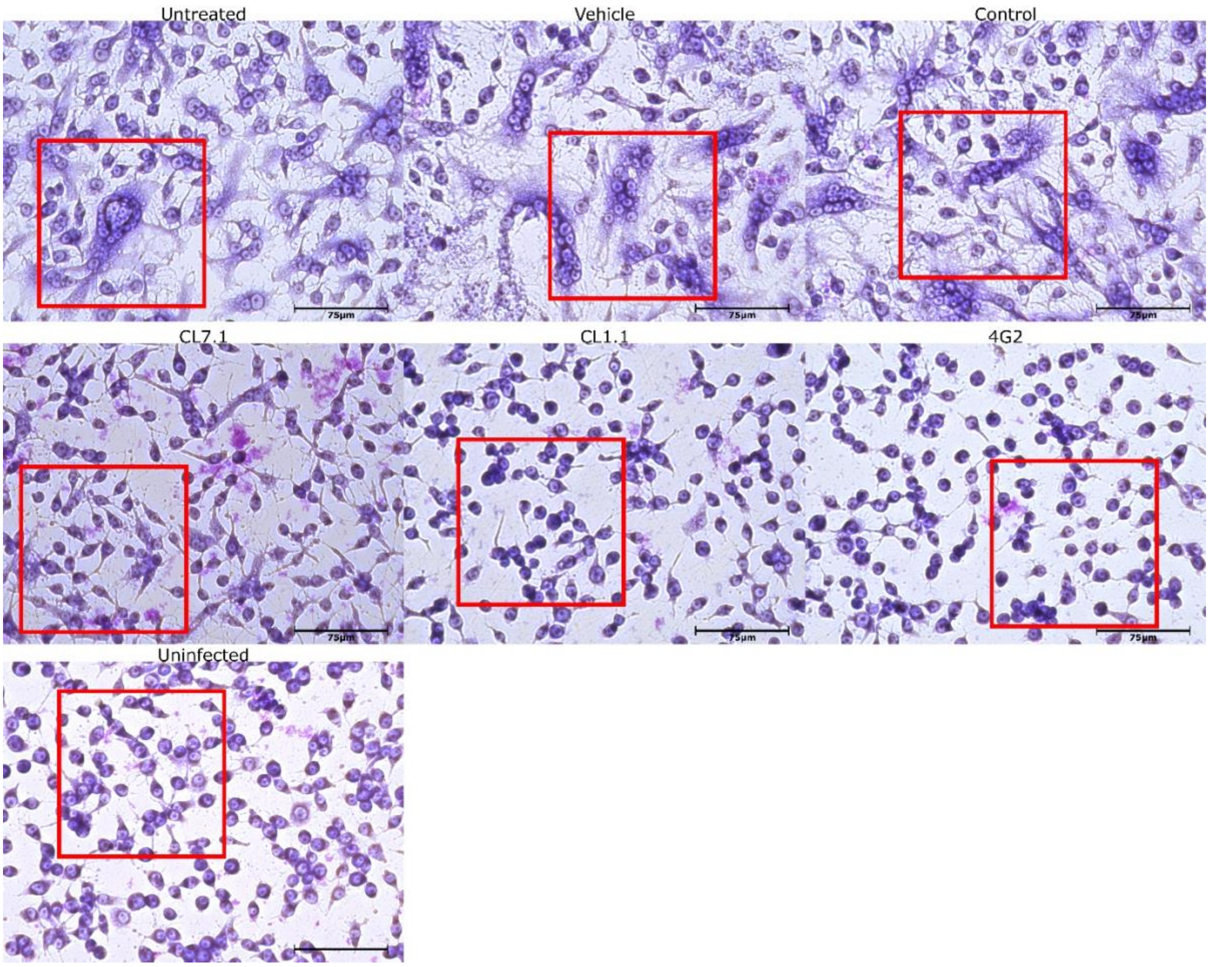

S7 Fig. Uncropped source pictures used for Fig. 3.

C6/36 cells were infected with ZIKV (MOI of 2) for 48 h prior to the experiment. The cells were then treated with 10 µM of CL1.1, 50 µM CL7.1 or 500 ng/mL 4G2 mAb for 1 h. Cell-to-cell fusion was then induced for 2 h. The cells were then fixed, stained, and analyzed with bright-field microscopy.

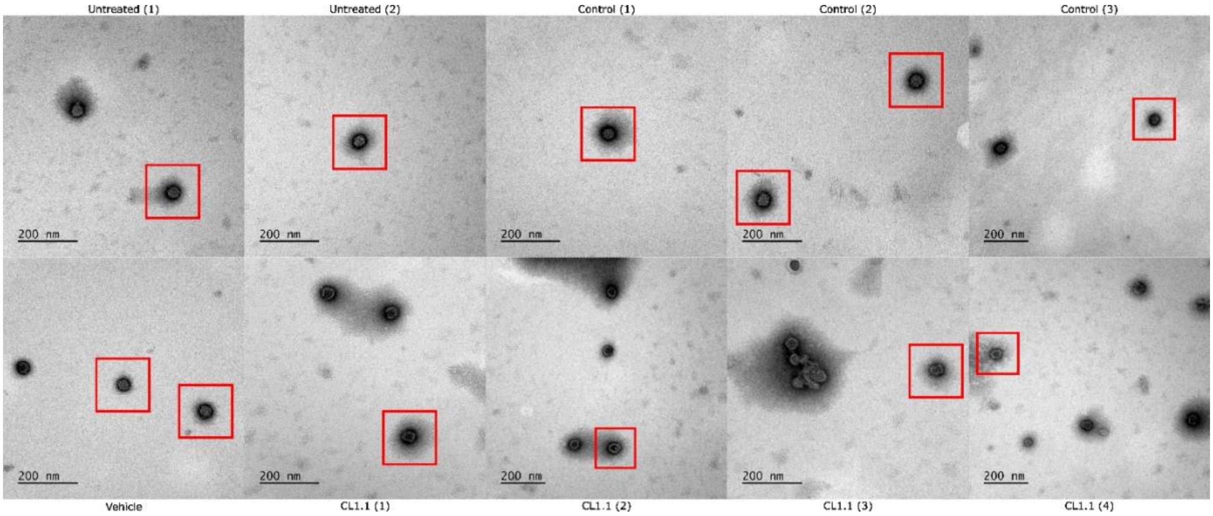

S8 Fig. Uncropped source pictures used for Fig. 5.

ZIKV (6.5×10^{10} PFU/mL, H/PF/2013 strain) was treated with 10 µM of CL1.1 for 1 h. The viruses were then analyzed by electron microscopy (compared to control peptide CL7.4 or vehicle alone).
